# The Role of Vitamin C, Vitamin D, and Selenium in Immune System against COVID-19

**DOI:** 10.3390/molecules25225346

**Published:** 2020-11-16

**Authors:** Minkyung Bae, Hyeyoung Kim

**Affiliations:** 1 Department of Food and Nutrition, Interdisciplinary Program in Senior Human Ecology, BK21 FOUR, College of Natural Sciences, Changwon National University, Changwon 51140, Korea; mkbae@changwon.ac.kr; 2 Department of Food and Nutrition, BK21 FOUR, College of Human Ecology, Yonsei University, Seoul 03722, Korea

**Keywords:** COVID-19, infectious disease, selenium, virus, vitamin C, vitamin D

## Abstract

Low levels of micronutrients have been associated with adverse clinical outcomes during viral infections. Therefore, to maximize the nutritional defense against infections, a daily allowance of vitamins and trace elements for malnourished patients at risk of or diagnosed with coronavirus disease 2019 (COVID-19) may be beneficial. Recent studies on COVID-19 patients have shown that vitamin D and selenium deficiencies are evident in patients with acute respiratory tract infections. Vitamin D improves the physical barrier against viruses and stimulates the production of antimicrobial peptides. It may prevent cytokine storms by decreasing the production of inflammatory cytokines. Selenium enhances the function of cytotoxic effector cells. Furthermore, selenium is important for maintaining T cell maturation and functions, as well as for T cell-dependent antibody production. Vitamin C is considered an antiviral agent as it increases immunity. Administration of vitamin C increased the survival rate of COVID-19 patients by attenuating excessive activation of the immune response. Vitamin C increases antiviral cytokines and free radical formation, decreasing viral yield. It also attenuates excessive inflammatory responses and hyperactivation of immune cells. In this mini-review, the roles of vitamin C, vitamin D, and selenium in the immune system are discussed in relation to COVID-19.

## 1. Introduction

Coronavirus disease 2019 (COVID-19) is caused by the severe acute respiratory syndrome coronavirus 2 (SARS-CoV-2). COVID-19 has spread rapidly across the world, with 39,944,882 confirmed cases and 1,111,998 deaths reported to the World Health Organization (WHO), as of 19 October 2020 [1]. It has been reported that the severity of COVID-19 can be influenced by various factors such as age, sex, ethnicity, and underlying comorbidities [2,3,4,5]. Although many therapeutic treatments have been suggested, there is no approved antiviral treatment specific for COVID-19.

Recently, the European Society for Clinical Nutrition and Metabolism (ESPEN) proposed 10 practical recommendations for the management of COVID-19 patients [6]. The recommendations include the prevention of malnutrition by providing adequate amounts of macronutrients to maintain energy, protein, fat, and carbohydrate requirements. Moreover, sufficient supplementation with vitamins and minerals is important for the prevention of viral infection.

Low levels of micronutrients such as vitamins A, E, B_6_, B_12_, zinc, and selenium have been associated with adverse clinical outcomes during viral infections [7]. A recent review by Zhang and Liu [8] demonstrated that besides vitamins A and D, vitamin B, vitamin C, omega-3 polyunsaturated fatty acids, and trace elements (selenium, zinc, and iron) should be considered in the assessment of micronutrients in COVID-19 patients. A recent small-scale nutritional status study on COVID-19 patients in Korea showed significant deficiency of vitamin D and selenium in patients with and without pneumonia [9]. Serum levels of vitamins B_1_, B_6_, B_12_, D, folate, selenium, and zinc were determined in 50 patients with COVID-19. Seventy-six percent of the patients had vitamin D deficiency, and 42% had selenium deficiency [9]. Vitamin D deficiency has been associated with a number of different viral diseases, including influenza [10,11,12] and hepatitis C [13]. However, other studies have questioned this relationship for influenza [14,15]. Selenium deficiency is associated with mortality in COVID-19 [16], and a sufficient selenium level is important for recovery from the disease [17]. Vitamin C is known to have antiviral effects, and a high-dose treatment shows beneficial effects in COVID-19 patients [18,19].

This mini-review discusses the role of vitamin C, vitamin D, and selenium in immunity, and the beneficial effects of these micronutrients in reducing the risk of infectious diseases, particularly COVID-19.

## 2. Micronutrients and the Immune System

### 2.1. The Role of Vitamin C in the Immune System

COVID-19 can develop into acute respiratory distress syndrome, secondary infection, and sepsis [20]. An intravenous treatment with high-dose vitamin C has shown beneficial effects on sepsis and septic shock [18,21]. An intravenous infusion of vitamin C (50 mg/kg body weight) every 6 h for 96 h significantly decreased mortality and increased the number of intensive care unit (ICU)-free days in patients with sepsis and acute respiratory distress syndrome, compared to the control group [19]. In another study, seven months of treatment with intravenous vitamin C, hydrocortisone, and thiamine significantly decreased hospital mortality in septic patients, compared to the control group. In addition, the treatment group had no sepsis-related progressive organ failure, including acute kidney injury [21]. In another case, a 74-year-old woman with COVID-19 developed acute respiratory distress syndrome and septic shock. The patient was treated with high-dose intravenous vitamin C (11 g/d for 10 d) and showed rapid recovery [19]. 

Vitamin C treatment has antiviral effects. Clinical trials have shown that administration of high doses of vitamin C has beneficial effects against the common cold [22,23]. A high-dose vitamin C (hourly doses of 1000 mg of vitamin C for the first 6 h and then 3 times daily for 3 d) treatment decreased flu and cold symptoms in patients when compared to the control group [22]. A meta-analysis has shown that administration of high doses of vitamin C at the onset of the common cold decreased the duration of the cold and relieved the symptoms, such as chest pain, fever, and chills [23]. An animal study has demonstrated that vitamin C treatment enhances resistance to viral infection. In this study, vitamin C supplementation in drinking water, as 3.3 g/L of sodium L-ascorbate, improved antiviral immune response at the early stage of viral infection, in the lungs of vitamin C-insufficient Gulo (−/−) mice infected with influenza A virus (H3N2/Hong Kong). The administration of vitamin C markedly improved the survival rate, with no death for 7 d, while all vitamin C-insufficient Gulo (−/−) mice infected with influenza A virus died within a week. Moreover, vitamin C supplementation increased interferon-α/β (IFN-α/β) in the lungs of Gulo (−/−) mice infected with influenza A virus, but did not change the levels of inflammatory cytokines, including interleukin 1α/β (IL-1α/β) and tumor necrosis factor-α (TNF-α), in the lungs of the mice [24]. Vitamin C and dehydroascorbic acid decreased the yield of influenza virus type A in Madin–Darby canine kidney (MDCK) cells derived from canine kidney cells in vitro [25]. The study suggests that the antiviral effect of vitamin C might be mediated by free radical formation or its binding to the virus or molecules involved in viral replication. Therefore, the antiviral effect of vitamin C may be attributed to the production of antiviral cytokines (IFN-α/β), free radical formation, or direct binding to the virus.

Vitamin C potentially attenuates excessive immune response in patients with COVID-19. Severe COVID-19 infection induces pulmonary and systemic inflammatory responses [26]. The microbial infection causes excessive activation of macrophages for production of inflammatory mediators and nitric oxide (NO) [27], which can be reinforced by oxidative stress and NO itself [28]. COVID-19 patients had significantly higher levels of molecules related to inflammation, such as NO_2_^−^, NO_3_^−^, C-reactive protein, and lactate dehydrogenase in blood, compared to healthy individuals. After oral or intravenous administration of vitamin C with methylene blue and a known antioxidant N-acetyl cysteine, the blood levels of NO_3_^−^, methemoglobin, C-reactive protein, and lactate dehydrogenase were markedly decreased in four out of five patients [28]. This study also demonstrated that pro-oxidant/antioxidant imbalance is present in patients with COVID-19 [28]. In another study, intravenous vitamin C was administered at a dose of 1 g every 8 h for 3 d, to 17 patients infected with COVID-19. After vitamin C treatment, the patients had decreased inflammatory markers, such as ferritin and D-dimer, and a fraction of the earlier inspired oxygen requirements [29]. These studies suggest that the administration of vitamin C may increase the survival rate in COVID-19 patients, by attenuating excessive activation of immune responses.

Vitamin C may prevent the hyperactivation of immune cells by inhibiting glyceraldehyde 3-phosphate dehydrogenase (GAPDH). The glycolytic enzyme GAPDH can regulate the rate of glycolysis in activated myeloid and lymphoid cells [30]. Vitamin C can be oxidized intracellularly and extracellularly into its inactive form dehydroascorbate [31]. Inside cells, dehydroascorbate is reduced to ascorbate, while reduced glutathione (GSH) is oxidized [32]. Oxidized glutathione (glutathione disulfide) can be reduced to GSH by nicotinamide adenine dinucleotide phosphate (NADPH) [32]. Vitamin C has antioxidant capacity; however, high-dose vitamin C can display pro-oxidant activity by decreasing reactive oxygen species (ROS) scavenging systems, including GSH and NADPH [33]. Increased ROS can induce DNA damage, followed by the activation of poly(ADP-ribose) polymerase (PARP) [34]. PARP consumes NAD^+^ to synthesize poly(ADP-ribose) for DNA repair [35]. As NAD^+^ is needed for GAPDH activity, depletion of NAD^+^ decreases the enzymatic activity of GAPDH. Thus, the inhibition of GAPDH by a high dose of vitamin C may reduce the activation of immune cells by decreasing adenosine triphosphate (ATP) production in the cells [30].

Clinical trials are needed to investigate the effect of vitamin C on COVID-19 infection. A clinical trial was conducted from 14 February, 2020 to 30 September, 2020 in Zhongnan Hospital of Wuhan University, China [36]. In the trial, 12 g of vitamin C in 50 mL of sterile water was administered to patients for 4 h, and this was repeated every 12 h; therefore, the total amount of vitamin C administered to each patient was 24 g/d. This study is one of the first clinical trials to administer intravenous vitamin C to treat COVID-19. The study will investigate whether intravenous vitamin C could suppress cytokine storms caused by COVID-19, improve pulmonary function, and reduce the risk of acute respiratory distress syndrome in COVID-19.

### 2.2. The Role of Vitamin D in the Immune System

Adequate vitamin D levels in the body can be achieved by sufficient vitamin D consumption and sun exposure. The risk factors for vitamin D deficiency are age, smoking, obesity, and chronic diseases such as diabetes and hypertension [37]. 25-hydroxyvitamin D levels were inversely correlated with acute respiratory infection, as reported in the National Health and Nutrition Survey (NHANES) 2001–2006 [38]. Sufficient concentration of 25-hydroxyvitamin D was associated with a reduction in the risk of acute respiratory tract infections in adults [39]. In addition, sufficient levels of 25-hydroxyvitamin D in the serum were inversely correlated with the risk of viral respiratory tract infection in children [40]. Moreover, there was a small case study conducted with 10 COVID-19 patients in Indonesia [41]. On blood analysis, nine patients had vitamin D deficiency, and one patient had insufficient levels of vitamin D. Therefore, there was no patient with adequate vitamin D levels in the study. This indicates that vitamin D deficiency might be a risk factor for viral infection.

Studies have investigated the relationship between vitamin D deficiency and COVID-19 [42,43]. As vitamin D can be synthesized by sunlight exposure on the skin, living at a higher latitude is a risk factor for vitamin D deficiency [44]. The prevalence rate of COVID-19 and the rate of related deaths were significantly higher in high-latitude states (latitude ≥ 37°) than in low-latitude states (latitude < 37°) in the United States [42]. The average annual hours of sunlight exposure was negatively correlated with COVID-19 mortality [45]. It has also been suggested that vitamin D deficiency (serum 25-hydroxy vitamin D level of 20 ng/mL or lower) and insufficiency (serum 25-hydroxy vitamin D level of 21 to 29 ng/mL) are associated with increased mortality in COVID-19 in Indonesia [43]. A meta-analysis of 11 studies covering 360,972 COVID-19 patients was conducted. Among the patients, 37.7% had vitamin D deficiency, and 32.2% had vitamin D insufficiency. Furthermore, the risk of COVID-19 was significantly increased in patients with low levels of vitamin D [46]. Another meta-analysis that covered 1368 COVID-19 patients showed that a low level of vitamin D was significantly associated with worse prognoses in the patients [47]. The mortality rate of hospitalized COVID-19 patients who were vitamin D sufficient (serum 25-hydroxy vitamin D ≥ 30 ng/mL) was 5%; however, patients with severe vitamin D deficiency (serum 25-hydroxy vitamin D < 10 ng/mL) had a 50% mortality rate after 10 d of hospitalization [48]. Vitamin D deficiency was positively correlated with hospitalization within 24 h and ICU admission during hospitalization in COVID-19 patients [49]. The aforementioned studies suggest that vitamin D deficiency can result in poor prognoses in patients with COVID-19. Therefore, vitamin D may be used as an adjunctive therapy for COVID-19 patients.

Vitamin D reduces the risk of viral infections. It improves the body’s physical barrier by regulating the production of proteins for tight junctions [50], adherens junctions [51], and gap junctions [52], which can be disturbed by infection by microorganisms, including viruses [53]. In addition, lung epithelial cells express 1α-hydroxylase that converts 25-hydroxyvitamin D3 to 1,25-dihydroxyvitamin D3, the active form of vitamin D [54]. Active vitamin D increases the expression of vitamin D-regulated genes, such as cathelicidin and toll-like receptor co-receptor CD14 in human tracheobronchial epithelial cells [54]. Double-stranded RNA produced by most viruses can increase the expression of 1α-hydroxylase, leading to increased production of active vitamin D and the expression of cathelicidin in human tracheobronchial epithelial cells [54]. Therefore, adequate vitamin D might prevent the invasion of coronaviruses by enhancing physical barriers and increasing the production of antimicrobial peptides in the lung epithelium.

Vitamin D stimulates the production of antimicrobial peptides, such as cathelicidin and defensins [55] that have antimicrobial activities against various microorganisms, including bacteria, viruses, and fungi [56,57]. In a study in mice, cathelicidin LL-37 decreased influenza A virus replication [58]. Human cathelicidin displays antiviral effects by reducing viral particles produced by respiratory syncytial virus in epithelial cells, thus decreasing cell death in HEp-2 human epithelial cells [59]. Human β-defensin 2 displays antiviral activity by destabilizing the viral envelope in respiratory syncytial virus, which inhibits its infection in human lung epithelial cells [60]. Thus, it is important to maintain sufficient vitamin D levels to produce antimicrobial peptides.

Vitamin D modulates helper T cell responses. It reduces T helper type 1 (Th1) immune responses [61] and induces Th2 responses [62]. Th1 cells produce pro-inflammatory cytokines, such as IFN-γ and TNF-β, while Th2 cells produce IL-4, IL-5, IL-10, and IL-13 [63]. As vitamin D induces a shift from Th1 to Th2 phenotype, it decreases Th1 cytokines but increases Th2 cytokines [64].

Vitamin D may prevent cytokine storms in patients with COVID-19. COVID-19 can lead to cytokine storms and immunogenic damage to the endothelium and alveolar membrane [36], which may contribute to mortality in COVID-19 [65]. Severely ill patients with COVID-19 have a high level of pro-inflammatory cytokines, such as IL-6, compared to patients with moderate symptoms [65]. The increased level of IL-6 in critically ill COVID-19 patients was related to the detection of SARS-CoV-2 nucleic acid in serum [66]. Vitamin D can decrease the production of pro-inflammatory cytokines, such as TNF-α, IL-6, IL-1β [67], IL-12, and IFN-γ [68]. The anti-inflammatory effect of vitamin D might be due to the inhibition of nuclear factor κB (NF-κB) activation [69]. The vitamin D receptor interacts with inhibitor of κB (IκB) kinase β to inhibit NF-κB activation, and the interaction is enhanced by vitamin D [69].

Recent studies have shown that vitamin D deficiency is correlated with poor prognosis in COVID-19 patients. However, there was no association between blood concentration of vitamin D and the risk of COVID-19 in the UK biobank [70]. Therefore, large-scale controlled studies are necessary to determine the effect of vitamin D on COVID-19.

### 2.3. The Role of Selenium in the Immune System

Selenium deficiency may be a risk factor for COVID-19 mortality. A cross-sectional study conducted in Germany showed that the serum level of selenium was significantly higher in the surviving patients with COVID-19 compared to the deceased patients with COVID-19 [16]. Another study also determined that the recovery rate from COVID-19 was significantly associated with selenium levels in patients in China [17].

Selenium deficiency exacerbates virulence and progression of viral infections, such as influenza A [71,72] and Coxsackievirus B3 [73]. Selenium-deficient mice developed more severe lung pathology due to influenza virus infection than selenium-adequate mice [72]. The virus isolated from the lungs of selenium-deficient mice at 5 d after infection had mutations in its genome that made it more virulent [72]. In another study, Coxsackievirus B3, a normally avirulent phenotype, resulted in heart damage in selenium-deficient mice. Coxsackievirus B3 underwent a genetic mutation to a virulent phenotype in the selenium-deficient mice [73]. Selenium deficiency in the host affects the viral genome, leading to the virus becoming more virulent.

Selenium demonstrates antiviral effect by regulating CD4^+^ T cell response. It increased CD4^+^ T cell activation, proliferation, and differentiation by maintaining the intracellular level of free thiol in mice fed with a high-selenium diet (1.0 mg/kg body weight) compared to a low-selenium diet (0.08 mg/kg body weight) or medium-selenium diet (0.25 mg/kg body weight) for 8 w [74]. CD4^+^ T cells isolated from mice fed with a high-selenium diet demonstrated increased T cell receptor (TCR) signaling and TCR-stimulated IL-2 expression. In addition, high consumption of selenium induced the Th1 phenotype with increased IFN-γ in CD4^+^ T cells [74]. Selenium deficiency induced severe interstitial pneumonitis in mice infected with influenza virus, when compared to selenium-adequate mice [71]. Selenium deficiency decreased the mRNA expression of IFN-γ and IL-2, but increased the mRNA expression of IL-10, IL-13, IL-4, and IL-5 in the mediastinal lymph nodes [71]. As IL-10, IL-13, IL-4, and IL-5 are part of Th2 responses, selenium deficiency may have induced more Th2 responses than Th1 responses in the lungs of mice infected with influenza virus.

Selenium is important for the function of cytotoxic effector cells, such as CD8^+^ T cells and natural killer (NK) cells. TNF-α and IFN-γ have antiviral effects against influenza virus in CD8^+^ T cells [75]. Selenium supplementation increased the plasma levels of TNF-α and IFN-γ in mice infected with the influenza virus [76]. The number of CD8^+^ T cells was lower in the lungs of selenium-deficient mice than in selenium-sufficient mice [71]. Dietary supplementation with selenium (200 μg/d for 8 w) increased the cytotoxicity of CD8^+^ T cells by increasing the number of cells in the human peripheral blood lymphocyte population [77]. Furthermore, selenium supplementation increased the lytic activity of NK cells from human peripheral blood lymphocytes [77] and mouse spleen [78]. Therefore, selenium supplementation may enhance the function of cytotoxic effector cells in COVID-19.

Selenium plays an important role in the production of antibodies. Selenoprotein deficiency induced impaired T cell maturation, functions, and T cell-dependent antibody response in mice [79]. Selenoprotein synthesis requires selenocysteine tRNA, for inserting the selenocysteine into the protein [80]. T cells deficient in selenocysteine tRNA displayed selenoprotein deficiency, and the selenoprotein-deficient T cells showed markedly reduced proliferation in response to T cell receptor stimulation [80]. Moreover, the serum levels of antibodies, such as immunoglobulin M (IgM), IgG1, IgG2a, IgG2b, and IgG3, were lower in selenoprotein-deficient mice than in control mice [80].

Blood coagulation may increase mortality in patients with COVID-19 [81]. A low plasma selenium concentration was correlated with increased tissue damage, presence of infection, and organ failure, as well as increased mortality in ICU patients. In addition, the plasma selenium level was positively correlated with minimum platelet count, minimum plasma antithrombin activity, and protein C activity in the patients [82]. Venous thromboembolism includes deep vein thrombosis and pulmonary embolism, which are commonly developed in critically ill patients with infection [83]. It has been reported that venous thromboembolism occurred in 27% of COVID-19 patients in the ICU [84]. In another report, the cumulative incidence of venous thromboembolisms in COVID-19 patients at 7, 14, and 21 d after ICU admission was 26%, 47%, and 59%, respectively. The incidences were significantly higher in ICU patients than in patients in the general wards [85]. Selenium deficiency increased the ratio of thromboxane A_2_ to prostacyclin I_2_ in rats [86], inducing vasoconstriction and blood coagulation [87]. This might explain the mechanism for venous thromboembolism in selenium-deficient COVID-19 patients. Further clinical trials are required to evaluate the beneficial effects of selenium against COVID-19 

## 3. Conclusions

Nutritional therapy should be a part of patient care for survival of this life-threatening disease (COVID-19), as well as for better and shorter recovery. Most importantly, checking malnutrition and providing optimal nutritional supplementation are critical steps for optimal functioning of the immune system in the human body. Patients with malnutrition are more likely to be from lower socioeconomic groups; thus, nutrition supplementation is important for the risk group as well as older adults who have a relatively weak immune system. In this review, we focused on the importance of vitamin C, vitamin D, and selenium for immunity enhancement. The immunomodulatory properties and the consequences of deficiencies or supplementation of these micronutrients against viral infectious diseases, including COVID-19, are summarized in Table 1. Since severely ill COVID-19 patients were reported to be deficient in more than one nutrient, we suggest that nutritional deficiencies may favor the onset of COVID-19 and increase the severity of the disease. Combination of some of these micronutrients (vitamin C, vitamin D, and selenium) may help to boost the immune system, prevent virus spread, and reduce the disease progressing to severe stages.

## Figures and Tables

**Table 1:** The effect of micronutrients on the immune system against viral infectious diseases.

Micronutrient	Immunomodulatory Properties	Consequences of Deficiency/Effects of Supplementation in Infectious Diseases, including Coronavirus Disease 2019 (COVID-19)
Vitamin C	Increasing antiviral cytokines, such as interferon (IFN)-α/β [24] Increasing free radical formation to decrease viral yield [25] Attenuating excessive inflammatory response [27] Ameliorating hyperactivation of immune cells by altering energy metabolism [30]	Decreased flu or cold symptoms due to treatment with high dose of vitamin C [22,23] Decreased inflammatory mediators/ markers due to the administration of vitamin C in COVID-19 patients [28,29]
Vitamin D	Improving the physical barriers of the body by regulating the production of proteins for tight junctions [50], adherens junctions [51], and gap junctions [52] Stimulating the production of antimicrobial peptides, such as cathelicidin and defensins [55] Modulating T helper (Th) cell responses to induce a shift from Th1 to Th2 responses [61,62,64] Preventing cytokine storms by decreasing inflammatory cytokines [67,68] and nuclear factor κB (NF-κB) activation [69]	Inverse correlation between vitamin D level and viral respiratory tract infection [38,39,40] Vitamin D deficiency/insufficiency observed in patients with COVID-19 [41] Inverse correlation between COVID-19 mortality and sunlight exposure [45] or vitamin D level [43,48] Worse prognosis in COVID-19 patients with a low level of vitamin D [47,49]
Selenium	Preventing mutations in viral genome [71,72,73] Increasing CD4^+^ T cell activation, proliferation, and differentiation; inducing Th1 phenotype [74] Enhancing the function of cytotoxic effector cells by increasing the cytotoxicity of CD8^+^ T cells and lytic activity of natural killer (NK) cells [77] Maintaining T cell maturation and functions, including T cell-dependent antibody production [79,80] Preventing vasoconstriction and blood coagulation [87], which may increase COVID-19 mortality [81]	Higher selenium level in surviving COVID-19 patients compared to deceased patients [16] Higher recovery rate from COVID-19 in patients with higher selenium levels [17]

## Abbreviations

COVID-19	Coronavirus disease 2019
ESPEN	European Society for Clinical Nutrition and Metabolism
GAPDH	Glyceraldehyde 3-phosphate dehydrogenase
GSH	Reduced glutathione
ICU	Intensive care unit
IFN	Interferon
Ig	Immunoglobulin
IL	Interleukin
IκB	Inhibitor of κB
MDCK	Madin–Darby canine kidney cells
NADPH	Nicotinamide adenine dinucleotide phosphate
NF-κB	Nuclear factor κB
NHANES	National Health and Nutrition Survey
NK	Natural killer cells
NO	Nitric oxide
PARP	Poly(ADP-ribose) polymerase
ROS	Reactive oxygen species
TCR	T cell receptor
Th	T helper type
TNF	Tumor necrosis factor
